# A Bioinspired Multimodal CNN-LSTM Network for EEG Analysis of Patients in Coma

**DOI:** 10.3390/s25226981

**Published:** 2025-11-15

**Authors:** Sérgio Baldo-Júnior, Murillo G. Carneiro, João L. M. Barbosa, Liang Zhao, João Batista Destro-Filho, Marcos Campos, Renato Tinós

**Affiliations:** 1 Department of Computing and Mathematics, University of São Paulo, Ribeirão Preto 14040-901, Brazil; sergiobaldo@usp.br (S.B.-J.); zhao@usp.br (L.Z.); 2 Faculty of Computing, Federal University of Uberlândia, Uberlândia 38400-902, Brazil; 3 Faculty of Electrical Engineering, Federal University of Uberlândia, Uberlândia 38400-902, Brazil; joao.maximiano@ufu.br (J.L.M.B.); jbdestro@ufu.br (J.B.D.-F.); 4 Uberlândia Clinical Hospital, Federal University of Uberlândia, Uberlândia 38405-320, Brazil; marcos.campos@ufu.br

**Keywords:** electroencephalogram, coma outcome, multimodal learning, convolutional neural networks, long short-term memory, genetic algorithms

## Abstract

Electroencephalography (EEG) is widely used for diagnosis and evaluation of neurological diseases, despite challenges from its high-dimensional and noisy temporal data, which complicate accurate brain signal classification. This study proposes a multimodal deep learning model combining Convolutional Neural Network (CNN) and Long Short-Term Memory (LSTM) layers to classify EEG signals, integrating patient information as additional modalities. CNN layers effectively extract spatial features and reduce EEG data dimensionality, while LSTM layers capture long-term temporal dependencies. A Genetic Algorithm (GA) selects relevant multimodal features and optimizes CNN-LSTM hyperparameters. The model was applied to outcome classification in comatose patients, achieving improved classifier performance compared to unimodal approaches. Experimental results demonstrate that multimodal integration and GA optimization significantly enhance accuracy, robustness, and generalization. The architecture shows promise for broader EEG classification tasks, potentially advancing clinical decision support based on EEG signals.

## 1. Introduction

Electroencephalography (EEG) is typically a cheap and noninvasive technique that measures electrical activity in the brain using electrodes attached to the scalp. The electrodes capture the summed electrical activity produced by groups of neurons in the cerebral cortex [1]. An EEG recording provides valuable insights for monitoring and diagnosing various neurological diseases and conditions, such as Alzheimer’s disease [2], Parkinson’s disease [3], epilepsy [4], sleep disorders [5], and coma [6]. Through analysis of EEG patterns, both normal and abnormal brain activities can be identified, aiding doctors in identifying neurological disorders.

Despite the satisfactory performance achieved by traditional machine learning methods in classifying EEG signals [7,8,9], a significant challenge lies in extracting relevant features to be used as inputs for these algorithms. Understanding the essential features that enable machine learning algorithms to classify EEG signals is crucial. Manual feature extraction methods may lack the necessary accuracy due to the inherent variability of EEG signal recordings.

Artificial Neural Network models (ANNs), particularly those based on Convolutional Neural Networks (CNNs) and/or Recurrent Neural Networks (RNNs), have been used for classifying EEG signals [10,11,12]. In contrast to traditional machine learning approaches based on predefined features, deep learning algorithms can automatically extract features from raw EEG data. RNNs have demonstrated their efficacy in capturing temporal information, making them useful in various medical applications. Unlike conventional RNNs, Long Short-Term Memory (LSTM) networks can handle long time-series data [13].

In this paper, we propose a new multimodal hybrid deep learning model to classify EEG signals named CNN-LSTM-GA. The proposed model is used to aid the outcome prediction of patients in coma. Coma can be caused by various health conditions [14]. Accurately predicting patient outcomes is vital for clinical decision-making and providing timely interventions [15,16]. In addition, outcome prediction enables optimizing treatment at the Intensive Care Unit (ICU), leading to a better work division within the health staff, as well as improving the preparation of possible organ donations.

The proposed hybrid architecture is defined by the combination of CNN and LSTM networks. The CNN layers provide more efficient representations of the EEG signals while reducing their high-dimensional space. On the other hand, the LSTM layers capture longer temporal dependence over sequential data. Additionally, the expressiveness of our hybrid architecture is increased by its ability to learn from multimodalities, such as those related to patient information or extracted from EEG signals by using predefined filters. Our hypothesis is that the predictive performance of CNN-LSTM-GA models is improved when using multimodal information, such as raw EEG signals and patient features obtained from clinical records.

Using CNN combined with LSTM allows the network to perform well on both spatial and temporal data. On the other hand, it represents a more complex technique in relation to the configuration of hyperparameters and architecture. In addition, the adoption of different sources of information increases the number of features, which does not necessarily mean an improvement in predictive performance. To handle this problem, we propose a Genetic Algorithm (GA) to optimize hyperparameters for the hybrid CNN-LSTM, and also to select the most important features. Experiments with hundreds of EEG data collected from 60 comatose patients at a public hospital were conducted in order to evaluate the CNN-LSTM-GA architecture, the strategies to consider additional modalities of information, and the optimization process conducted by GA. In summary, the main contributions of this manuscript are listed as follows: 

- A multimodal CNN-LSTM-GA model is proposed to predict the outcome of patients in coma from their EEG records, while CNN layers extract features from the EEG records, the LSTM layers capture relationships over temporal sequences.
- A GA is proposed for the selection of the most important features and to optimize the hyperparameters of the proposed model.
- The proposed model is able to learn from other modalities of information besides the EEG signals, such as those related to patient information (e.g., sex and age). A fully connected layer fuses the outputs of the LSTM layer and the selected features from other modalities.

The multimodal CNN-LSTM-GA architecture proposed is a novel approach aimed at significantly improving the outcome prediction of comatose patients. By enhancing the accuracy of outcome predictions, more informed clinical decisions and timely interventions can be taken for better patient care, also including the improvement of work organization within the ICU and more efficient possible organ donations.

Furthermore, the proposed GA extends beyond conventional hyperparameter tuning or feature selection approaches. It performs a joint optimization of the CNN–LSTM architecture and multimodal feature subsets within a framework that balances predictive performance and model complexity. Unlike regularization-based or cross-validation strategies, which primarily adjust model parameters or assess robustness, the GA explores the global search space by evolving both the structure and parameters of the network. Although this process introduces some computational cost, it enables a broader and more effective exploration of the multimodal feature space, leading to improved predictive performance and more insights regarding interpretability.

The remainder of this paper is organized as follows. Section 2 presents the main related works in the literature. Section 3 introduces the problem addressed by our investigation as well as the multimodal dataset considered. Section 4 describes the proposed CNN-LSTM-GA architecture and the optimization strategy developed to optimize hyperparameters and feature selection. Section 5 discusses the results of the proposed technique in comparison to other techniques, and presents the main insights obtained from the experiments. Section 6 concludes the paper.

## 2. Related Works

In this section, we present the main works related to our research. First, we provide a focused analysis of studies employing Convolutional Neural Networks (CNNs) for EEG signal classification. Next, we review approaches that combine CNNs with Long Short-Term Memory (LSTM) networks. Finally, we discuss recent advancements using Transformer-based models, which have shown promising results. Additionally, we examine EEG studies that incorporate different sources of information. We conclude by highlighting the main contributions of our study in comparison to the most related works in the literature.

The study by [17] presents STEADYNet, a lightweight convolutional neural network (CNN) architecture designed for the automatic detection of dementia-related conditions including Alzheimer’s disease (AD), Mild Cognitive Impairment (MCI), and Frontotemporal Dementia (FTD) using EEG signals. The model emphasizes both performance and simplicity, leveraging a low-complexity design that reduces computational cost and inference time without compromising accuracy. STEADYNet was evaluated on two publicly available multi-class datasets, demonstrating robust classification results with high accuracy, sensitivity, and specificity across all dementia classes. Additionally, the study employed a tailored adjustment of the Adam optimizer for each dataset. In contrast, the model proposed in our study focuses on the prognosis of patients using a multimodal architecture that integrates EEG signals with additional clinical data.

Xu et al. [12] proposed a deep learning model that combines CNN and LSTM networks to classify EEG signals. A public dataset with five types of EEG recordings was used to evaluate the model on two tasks: classification of seizure from non-seizure activity; and classification of EEG type. Ay et al. [10] proposed a hybrid model that combines CNNs and LSTM to detect depression. The EEG signals were used as input for the model to classify the presence of depression in patients. Lee et al. [11] employed a CNN-LSTM model to identify Parkinson’s disease from EEG. Pandey et al. [18] used a deep learning model based on CNNs and LSTM for automated seizure detection.

Deep learning models have demonstrated significant potential in medical signal processing. However, researchers often face the challenge of finding the optimal hyperparameters that define the architecture and training of these models for specific tasks. For example, Xu et al. [12] explored different values for the number of neurons in the LSTM network to identify the configuration that produces the best model performance. Similarly, Ay et al. [10] employed a brute-force approach to select the best architecture from predefined configurations of the CNN-LSTM model. An interesting approach for optimizing the hyperparameters of ANNs is to use Metaheuristics. In particular, Genetic Algorithms (GAs) have been employed in various studies to optimize the architectures and other hyperparameters of ANNs [19,20,21].

As far as we know, most studies on deep learning algorithms for classifying EEG signals have not incorporated multimodal information, such as age, sex, and medical history, for decision-making purposes. However, medical experts generally consider several modalities of information during EEG analysis. For example, factors such as age and stroke history are crucial to predict a patient’s treatment outcome. In this sense,  Baldo-Júnior et al. [22] investigated an hybrid CNN-LSTM architecture to classify coma etiology from EEG signals. Outputs of the CNN-LSTM and additional features, such as age, sex and statistical measures are introduced as extra inputs to the first dense layer of the model. The best architecture achieved a macro F1 score of 0.60, highlighting the challenges posed by the EEG classification task, particularly due to noise and high-dimensional features. Beyond the difference in application focus (coma etiology vs. coma prognosis), that study also diverges from ours architecturally, as our method relies on a GA to configure the architecture, select features, and optimize hyperparameters.

Combining external information as additional features for decision-making using deep learning algorithms presents another challenge: selecting the most relevant information for the classifier. GAs have also been employed to address this task [23]. In the “wrapper” approach, each GA individual represents a subset of features using a binary chromosome, where each element indicates the presence (1) or absence (0) of a specific feature.

Del-Lama et al. [21] addressed some of these challenges in the context of medical images, including combining information from different sources, hyperparameter optimization, and feature selection. The study specifically focused on classifying Magnetic Resonance Images to detect fractured vertebral bodies. They proposed a hybrid deep learning model based on CNNs that incorporated features from three distinct sources: (i) intermediate layers of CNNs; (ii) radiomics; and (iii) additional clinical and image histogram information. External features were included as additional information in the first dense layer of a CNN. A GA was used for (i) selecting a relevant subset of radiomic, clinical, and histogram features for image classification; and (ii) optimizing hyperparameters for the CNN. Experimental results using various models demonstrated that combining information from different sources improved classifier performance.

The STRFLNet was proposed by [24], a spatio-temporal representation fusion learning network designed for emotion recognition using EEG signals. The approach was evaluated on three diverse datasets encompassing various emotional categories such as positive, neutral, negative, happiness, sadness, fear, and high- or low-arousal states. STRFLNet encodes the latent spatio-temporal dynamics of multi-channel EEG data through a multi-module architecture, consisting of the Temporal Transformer Encoder Module (TTEM), the Continuous Dynamic–Static Graph Ordinary Differential Equation Module (CDSGODEM), the Hierarchical Transformer Fusion Module (HTFM), and a Classification Module (CM). Notably, the introduced dynamic–static graph structure allows the model to capture both persistent and transient brain connectivity patterns associated with emotions, while the CDSGODEM models continuous spatial features that help overcome limitations like over-smoothing. Furthermore, the hierarchical fusion strategy within the HTFM enhances the integration of complementary multi-domain spatio-temporal features by emphasizing latent dependencies. Unlike STRFLNet, our proposed method leverages Genetic Algorithms for feature selection and hyperparameter optimization, targeting improved model performance within a clinical prognosis context.

Another study employing Transformers is EEGTransformer [25], which leverages the Transformer encoder’s power to extract complex spatio-temporal patterns from EEG signals for coma prognosis. This work uses the same dataset as the present study, consisting of EEG recordings from adult ICU patients. The model architecture includes a Transformer encoder module that captures long-range temporal and spatial dependencies across EEG channels, followed by a classification module. Extensive hyperparameter tuning was performed, adjusting Transformer layers, attention heads, intermediate layer size, and dropout rate to achieve optimized performance and reliable prognostic results.

Our work is inspired by [21] but follows a different approach that considers LSTM networks with CNNs, i.e., both CNNs and LSTM are combined into a unique architecture aiming to exploit the advantages of each of them. Other works [10,11,12,18] also investigated the combination of CNN and LSTM networks for the analysis of EEG signals. Our work differs from other approaches by developing a CNN-LSTM architecture designed to learn from different modalities of information, like EEG signals and patient clinical records, and by using a GA to select hyperparameters and features.

The proposed approach is evaluated in the prediction of the outcome of patients in coma. The dataset considered in this work is a modified version of that adopted in Ramos and Destro-Filho [26], in which the authors aimed to provide a quantitative description of coherence values measured by EEG signals in Brazilian individuals. The analysis included several statistical comparisons to explore differences in coherence behavior based on levels of consciousness. Based on such a dataset, several quantitative descriptors were further considered in Carneiro et al. [27], which investigated techniques based on complex networks for the prognosis of patients in coma.

To better illustrate the techniques applied in each related work mentioned, we present Table 1, which offers a comprehensive comparison based on the key technologies employed. The table categorizes each study according to its use of deep learning (DL), hybrid deep learning (HDL), multimodal data integration (M), feature selection methods (FS), and hyperparameter optimization (HPO). This structured comparison not only highlights the diverse strategies found in the literature but also clearly demonstrates how our proposed model integrates these advanced features in a unique and comprehensive manner, underscoring its novelty and completeness within this research domain.

Our multimodal CNN-LSTM-GA architecture proposed in this article uniquely integrates a hybrid deep learning approach within a multimodal framework. As shown in Table 1, unlike previous studies, our work combines hybrid deep learning (HDL) techniques with multimodal data processing (M), while simultaneously employing a Genetic Algorithm (GA) for automatic feature selection (FS) and hyperparameter optimization (HPO). This comprehensive integration of multiple advanced techniques distinguishes our model as the most complete among related works, effectively enhancing model performance and adaptability in complex multimodal EEG analysis scenarios.

## 3. EEG Dataset and Feature Description

Predicting patient outcomes is essential for making clinical decisions and timely interventions. In this way, we consider the problem of outcome prediction of patients in coma, in which the model’s output corresponds to one of two categories: favorable or unfavorable outcome.

### 3.1. Dataset Description

The dataset is composed of information from 60 comatose patients who were admitted to the Clinical Hospital of the Federal University of Uberlândia (HC-UFU) between 2010 and 2014. The data were collected by the hospital’s technical team, responsible for comatose patients in the adult ICU, with the approval of the Ethics Committee of the Federal University of Uberlândia under protocol number 369/11.

The patients underwent an EEG recording session approximately 14 days after being admitted to the hospital. The EEG recording lasted approximately 20 min and was conducted using a 20-channel EEG cap with active electrodes and a BrainNet BNT-EEG device (Linx BNT^®^). The acquisition process involved placing 20 electrodes in specific regions of the cerebral cortex, following the international 10-20 system: FP1, FP2, F7, F3, FZ, F4, F8, T3, C3, CZ, C4, T4, T5, P3, PZ, P4, T6, O1, OZ, and O2. It is important to note that no external stimuli were presented to the patients during the recording sessions.

Additional information, including age, sex, coma etiology, and outcome, was collected from the clinical records of each individual. The average age of the patients was 49.16 ± 19.18, with 44 males and 16 females. Table 2 presents an overview of the number of patients for each coma etiology and their corresponding outcomes. A favorable outcome was defined as patient survival, while an unfavorable outcome was defined as patient death.

**Figure 1:**
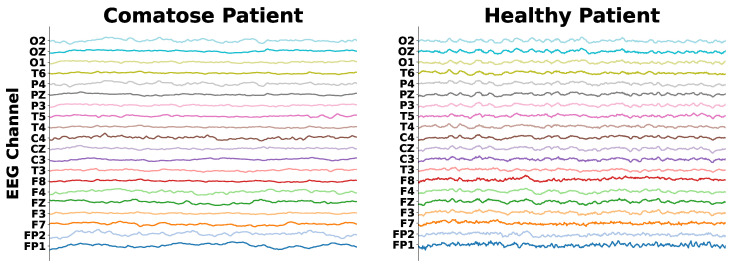
EEG signals of a comatose and a healthy patient.

Comatose patient’s EEG data are characterized by signal power concentrated at low frequencies (1–7 Hz, or delta and theta bands), throughout all cerebral regions, so that there is almost no signal power at high frequencies (above 7 Hz, like alpha and beta waves) [28]. Otherwise, normal individual EEG recordings present most of the signal power at high frequencies, with alpha waves lying at the posterior regions. This can be observed in Figure 1, which presents a comparison of EEG data collected from a comatose patient and a healthy control patient.

For each 20 min EEG session, 10 segments, each lasting 2 s, were extracted, resulting in a total of 10 segments’ samples (examples of the dataset) for each patient, which resulted in a dataset with a total of 600 segments’ samples. Each segment corresponds to a fraction of the EEG data captured by the 20 channels. The frequency range of the recordings varied between 100 and 600 Hz. As a result, the dataset comprises segments’ samples from different patients, each with its frequency range. No downsampling was performed to establish a standardized sampling rate. A team of clinical neurologists with extensive expertise conducted a detailed visual analysis of the EEG recordings and selected each one of the segments, ensuring minimal noise presence.

### 3.2. Feature Description

In the following, we describe both patient and time-domain features considered in this study: 

- *Patient Features*. The patient features, another modality of information, were extracted from the patient’s electronic medical records. They are (i) patient’s age; (ii) patient’s sex; (iii) coma etiology; and (iv) coma outcome.
- *Time-Domain (TD) Features*. We extracted time-domain features from each 2 s segment of the EEG data. As CNNs have the ability to automatically extract features from inputs, we opted to extract basic features directly from the signals. The selected TD features are (i) mean; (ii) minimum; (iii) maximum; (iv) standard deviation; and (v) variance. As every segment had 20 channels, and each channel captures its own signal, the total of TD features extracted from each segment is 100.

## 4. The Proposed Multimodal CNN-LSTM-GA

In this section, we present our multimodal CNN-LSTM architecture optimized by GA to aid medical experts in analyzing EEG signals. The model takes EEG signals as inputs and incorporates multimodal features, such as patient information from clinical records and features extracted from EEG signals, as additional inputs to the latent representation learned by the CNN-LSTM. In addition, we also designed a GA to optimize the subset of multimodal features from EEG signals and patient features, as well as to find the optimal hyperparameters of the CNN-LSTM model. We refer to our proposed architecture as CNN-LSTM-GA.

**Figure 2:**
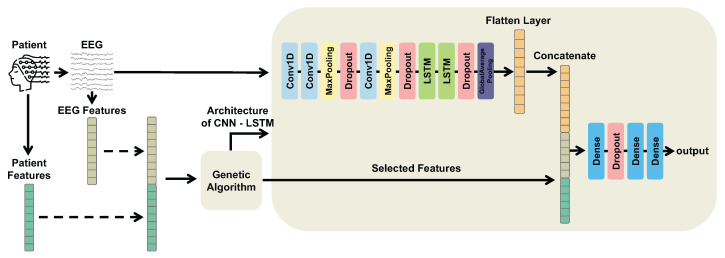
Overview of the proposed multimodal CNN-LSTM-GA architecture. Electroencephalogram (EEG) segments are processed through Convolutional Neural Network (CNN) layers followed by Long Short-Term Memory (LSTM) layers to extract temporal and spatial features, producing a latent representation. Patient features and time-domain EEG descriptors are then concatenated with this latent representation. A Genetic Algorithm (GA) is employed to select the optimal subset of multimodal features and to optimize the model architecture and hyperparameters.

A general overview of our multimodal CNN-LSTM-GA architecture is illustrated by Figure 2. CNN-LSTM-GA is able to process different modalities of information through a CNN-LSTM architecture. On the one hand, multimodal features aim to increase the expressiveness of our hybrid architecture by providing complementary information. On the other hand, CNN-LSTM architecture allows the model to capture both spatial and temporal aspects of the input data.

According to Figure 2, the multimodal information is processed as follows. On the one hand, EEG segments are input to the initial CNN layer and pass through a series of convolutional and pooling layers to automatically extract informative features for classification. These features are then fed into LSTM layers to capture temporal dependencies, and the LSTM output forms the latent representation. On the other hand, patient features from clinical records and TD features from EEG segments are fused with this latent representation to provide richer input to the fully connected layers.

To formally describe this fusion process, we now present the mathematical formulation, which constitutes a key component of our model.

Let $X_{EEG}\in R^{B\times T\times C}$ denote the EEG input, where *B* is the batch size, *T* is the number of time samples, and *C* is the number of EEG channels. Each EEG segment corresponds to a multichannel time series captured by 20 electrodes (C=20) with approximately T=2000 samples per segment.

The CNN-LSTM subnetwork first applies a sequence of one-dimensional convolutions and pooling operations to extract spatial patterns from the EEG input: 

(1) $$F_{CNN}=\mathrm{CNN}\left(X_{EEG}\right)\in R^{B\times T^{\prime }\times D_{CNN}},$$

where $T^{\prime }$ is the reduced temporal dimension after pooling operations, and $D_{CNN}$ is the number of convolutional filters (i.e., the depth of the CNN feature maps).

The feature maps $F_{CNN}$ are then processed by the LSTM layers to capture long-term temporal dependencies and produce a latent temporal representation: 

(2) $$H_{LSTM}=\mathrm{LSTM}\left(F_{CNN}\right)\in R^{B\times D_{LSTM}}.$$

where $D_{LSTM}$ is the dimensionality of the hidden state (i.e., the number of LSTM units).

Patient and time-domain (TD) features are represented as $X_{P}\in R^{B\times D_{P}}$ and $X_{TD}\in R^{B\times D_{TD}}$, respectively, where $D_{P}$ and $D_{TD}$, correspond to the patient variables and the time-domain descriptors extracted from each EEG segment selected in the GA. These two feature sets are first concatenated to form a single multimodal vector: 

(3) $$Z_{PTD}=\left[X_{P}\;∥\;X_{TD}\right]\in R^{B\times (D_{P}+D_{TD})}.$$

The resulting vector $Z_{PTD}$ is then concatenated with the latent EEG representation obtained from the LSTM: 

(4) $$Z_{fusion}=\left[H_{LSTM}\;∥\;Z_{PTD}\right]\in R^{B\times (D_{LSTM}+D_{P}+D_{TD})},$$

where ∥ denotes concatenation along the feature dimension. The fused multimodal representation is finally passed through fully connected layers to produce the classification output: 

(5) $$\hat{y}=\mathrm{Softmax}\left(f_{FC}\left(Z_{fusion}\right)\right).$$

This corresponds to a *hierarchical fusion strategy*, where the EEG signal is first encoded through CNN and LSTM layers to obtain a latent temporal–spatial representation $H_{LSTM}$, which is then combined with patient and TD embeddings after modality-specific processing. For the dataset used in this study, the resulting latent representation has dimension $H_{LSTM}\in R^{B\times 128}$ before fusion.

We also developed a GA to optimize the selection of multimodal information related to patient and TD features, as well as to find the optimal hyperparameters of the CNN-LSTM model. In a GA, a population of individuals or chromosomes, which represent potential solutions to a problem, undergoes selection, crossover, and mutation inspired by natural selection and genetics [21], in which each individual is evaluated by a fitness function able to define how good that corresponding solution is.

**Figure 3:**
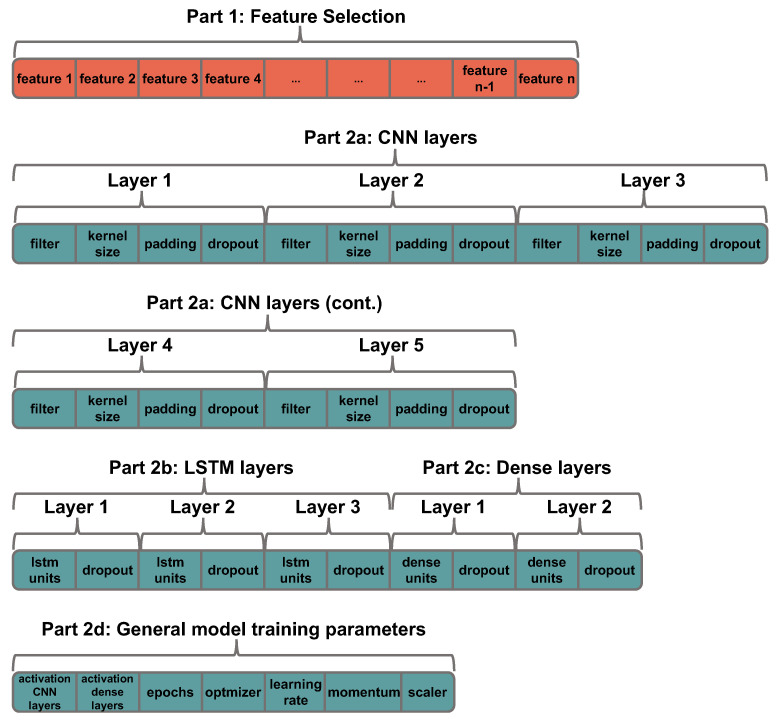
Representation of the solution (values for the hyperparameters of the CNN-LSTM and features) in the chromosome of the individual.

We designed an encoding in which each GA individual consists of two major parts: one for the feature selection (part 1) and one for optimization of the hyperparameters that define the CNN-LSTM architecture (part 2). Each GA individual encodes a subset of features (binary representation) and the hyperparameters of the CNN-LSTM (integer, real, or categorical values), forming a mixed-type optimization problem. Figure 3 shows how the feature selection strategy and the hyperparameter optimization of the CNN-LSTM are codified in the chromosome of a GA individual. In the part 1 (left side of an individual denoted by red color in the figure), each individual represents a specific subset of features, in which the *i*-th binary element of the chromosome indicates whether the *i*-th feature is included or excluded. In the part 2 (right side of an individual denoted by blue color in the figure), the hyperparameters to be optimized are divided in four groups related to CNN layers (part 2a), LSTM layers (part 2b), fully connected (dense) layers (part 2c), and general training parameters (part 2d). In addition, we also defined empirically that the GA-coded architectures have up to five convolution layers, up to three LSTM layers, and up to two dense layers, which is often enough to obtain good results. Table A1 of the Appendix A provides the range of hyperparameter values that can be selected by the GA at each position of the encoding.

**Figure 4:**
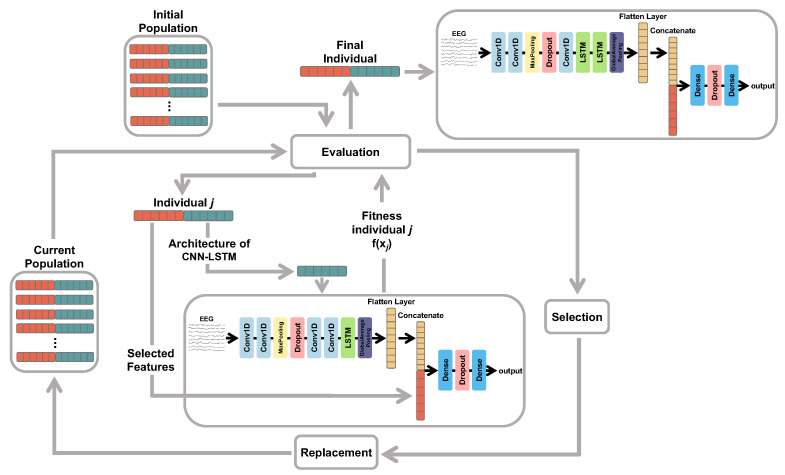
Flowchart of the GA utilized for feature selection and the selection of hyperparameters for the hybrid CNN-LSTM model.

The simplified flowchart of the proposed GA is presented in Figure 4. Firstly, an initial population of candidate solutions is randomly generated following the encoding presented in Figure 3. Each individual is then evaluated according to a fitness function in which its corresponding features and hyperparameters are considered. Genetic operators of selection and replacement (crossover and mutation) are applied in order to produce better configurations of candidate solutions. Such a procedure is repeated by a fixed number of generations (iterations). At the end, we expect to achieve an optimal configuration of multimodal features and hyperparameters able to provide good results for a given EEG classification task.

We defined the F1 score as the fitness function for each candidate solution. To obtain that, the dataset was divided into three distinct parts: 40% for training, 40% for validation, and 20% for testing. The stratified method was used, which ensures that the proportions of samples for each category are maintained when randomly partitioning the dataset. During the GA execution process, the training and validation parts were utilized. Each GA individual was evaluated by training the corresponding model using the training data, and the F1 score was calculated using the validation examples. At the end of the GA execution, the best individual was evaluated by retraining the model with both the training and validation sets and testing it using the test set.

Regarding the genetic operators, we designed selection and crossover as follows: selection adopts a comparison (tournament) between two individuals randomly selected from the population, in which the individuals with better fitness are considered for crossover; and crossover considers a slice of two points randomly defined to produce new individuals by swapping such chromossomes’ values between a given pair of selected individuals. Given that the individuals consist of two major parts, their chromosomes contain a variety of data types, including Boolean, integer, or string. Consequently, different types of mutations are implemented based on the specific data type found in the randomly chosen chromosome position undergoing mutation: 

- *Boolean mutation* occurs when a mutation affects a position on the chromosome where the data type is Boolean. This mutation involves flipping the value present in the original chromosome.
- *Window mutation* is applied when the mutation occurs at a chromosome position where the data type is integer or real. In this case, the values are ordered, and the mutation involves creating a subset considering the two values before (smaller) and after (bigger) the value present in the chromosome, if any. A new value is then randomly chosen in this subset of values.
- *Nominal mutation* is used when a mutation occurs in a chromosome position that represents a nominal categorical variable (string), meaning a variable with unordered values. In this case, a new value is randomly selected, from all possible values, to replace the value present in the chromosome. Notice that, in the case of nominal categorical variables with only two values, the nominal mutation is similar to the Boolean mutation.

The pseudocode describing the optimization process is presented in Algorithm 1. The complete implementation of the proposed CNN–LSTM–GA model, including data preprocessing, training, and evaluation procedures, is publicly available at our GitHubrepository (https://github.com/SergioBaldo/CNN-LSTM-GA, accessed on 22 September 2025).
**Algorithm 1** CNN-LSTM-GA: Feature selection and hyperparameter optimization**Input: ***D*—Dataset, $N_{pop}$—population size, $N_{gen}$—number of generations, $p_{c}$—crossover probability, $p_{m}$—mutation probability  **Output: ** Best individual $I^{*}$ and corresponding CNN-LSTM model $M^{*}$    1:Split *D* into train (40%), validation (40%) and test (20%) subsets;*// Initialize population P with $N_{pop}$ individuals*  2:**for** each individual i∈P **do**  3:      Randomly initialize feature subset $F_{i}$ and model hyperparameters $H_{i}$;  4:**end for**  5:**for** generation = 1 to $N_{gen}$ **do**  6:      **for** each individual i∈P **do**  7:            Decode chromosome → feature subset $F_{i}$ and model hyperparameters $H_{i}$;  8:            Build CNN-LSTM($H_{i}$);  9:            Construct $T_{train}$ and $T_{test}$ datasets from EEG data using feature subset $F_{i}$;10:            Train CNN-LSTM($H_{i}$) model with $T_{train}$;11:            Compute fitness f(i) as the F1-score of CNN_LSTM($H_{i}$) evaluated on $T_{validation}$;12:      **end for**13:      Apply tournament selection to choose parents based on f(i);14:      For each parent pair, generate two offspring using crossover with probability $p_{c}$.15:      **for** each offspring gene *g* with probability $p_{m}$ **do**16:            **if** *g* is Boolean **then**17:                  Flip gene *g*;18:            **else if** *g* is Integer or Real **then**19:                  Apply window mutation;20:            **else if** *g* is Nominal **then**21:                  Replace with random category;22:            **end if**23:      **end for**24:      Form new population *P* combining best individuals and offspring;25:**end for**26:Select best individual $I^{*}=\arg\max_{i}f\left(i\right)$;27:Decode individual $I^{*}$→ feature subset $F_{i}$ and model hyperparameters $H_{i}$;28:Build CNN-LSTM($H_{i}$);29:Construct $T_{train}$ and $T_{test}$ datasets from EEG data using feature subset $F_{i}$;30:Train CNN-LSTM($H_{i}$) model with $T_{train}$;31:Evaluate final model $M^{*}$ on $T_{test}$;32:**return** $I^{*},M^{*}$

## 5. Results and Discussion

The algorithms were implemented in Python (version 3.9.15) using the TensorFlow library (version 2.10.0). A workstation with an Intel@ Core i7-12700 Alder Lake processor (12 cores, 20 threads, 25 MB cache), 128 GB of RAM, and a GF AMPERE RTX 3060 12 GB 3584 CUDA GPU was used for running the codes. We compared the proposed model against several other models, considering monomodal and multimodal simulations. By comparing different models and modality variations, it is possible to evaluate the individual contribution of different mechanisms, e.g., to assess whether adding patient features is beneficial or not. The compared models with only a simple modality are: 

- *EEGTransformer [25]*: A TransformerEncoder based model designed to capture complex spatio-temporal patterns in raw EEG data, thereby enhancing class separability and prediction reliability.
- *MLP w/Patient Feats*: an MLP that uses only patient features as inputs. All MLP models were trained for 30 epochs, using the Adam optimizer with a learning rate of 0.0001, and the features were normalized using standard scaling. The number of neurons in the hidden layer was defined as the number of input features. The output layer adopts a sigmoid function with a single neuron, and the model was trained using the binary accuracy metric and the binary crossentropy loss;
- *MLP w/TD Feats*: an MLP that uses only TD features as inputs;
- *CNN-LSTM-baseline*: a CNN-LSTM model that uses only raw EEG signals as inputs to the CNN;
- *CNN-LSTM w/Patient Feats*: a CNN-LSTM model that uses EEG signals as inputs to the CNN and patient features as additional inputs to the first dense layer;
- *CNN-LSTM w/TD Feats*: a CNN-LSTM model that uses raw EEG signals as inputs to the CNN and time-domain features extracted from EEG signals as additional inputs to the first dense layer.

The multimodal models under comparison are: 

- *MLP w/Patient + TD Feats*: an MLP that uses patient and time-domain features extracted from EEG as inputs;
- *CNN-LSTM w/Patient + TD Feats*: a CNN-LSTM model that uses raw EEG signals as inputs to the CNN and patient and time-domain features extracted from EEG signal as additional inputs to the first dense layer;
- *CNN-LSTM-GA (Proposed)*: a CNN-LSTM model with the hyperparameters optimized by GA that uses EEG signals as inputs to the CNN and multimodal features selected by GA as additional inputs to the first dense layer (Figure 2). Table A2 of the Appendix A presents the architecture and the configuration of hyperparameters for CNN-LSTM-GA.

The number of runs of the GA was 10; in each run, a different pseudo-random seed was used. The population size was 30 individuals, and the number of generations was 30. The crossover rate was 0.6 and the mutation rate was $\frac{2}{n}$, where *n* is the size of the chromosome. F1 score (macro) was used as the fitness measure for evaluating the individuals.

### 5.1. Evaluation Procedures

Two evaluation procedures have been defined to our experiments as follows: 

- *Hold-out.* In order to simulate a real-world scenario, we randomly divided the dataset into 40% for training, 40% for validation, and 20% for testing.
- *Cross-validation (CV).* We also employed a stratified five-fold cross-validation approach where the dataset was randomly partitioned based on patients. The patients were divided into five equal-sized groups. During each iteration, one group was designated as the test set, while the remaining groups were used for training.

Since there are ten segments’ samples per patient, in both hold-out and CV experiments we only utilized the first nine segments for our tests, i.e., the classification was performed on these nine segments’ samples, and the algorithm’s final prediction for each patient was determined by selecting the most frequent classified category.

For the evaluation of EEG classification models, metrics that capture both overall accuracy and class-specific performance are essential, particularly due to the common presence of imbalanced class distributions. Accuracy provides a general overview of correct predictions; however, it may be misleading when classes are not evenly represented. Therefore, precision, also known as positive predictive value, measures the proportion of true positive predictions within all positive predictions for a class, while recall (sensitivity) reflects the proportion of actual positive cases correctly identified by the model. Additionally, the F1-score, as the harmonic mean of precision and recall, offers a balanced performance metric at the class level, especially valuable in imbalanced scenarios. These metrics derive from the confusion matrix, which displays counts of true positives, true negatives, false positives, and false negatives, as illustrated in Table 3. The corresponding equations are detailed in Table 4.

### 5.2. Prediction of Coma Outcome

In this experiment, the model’s output corresponds to either a “favorable” or an “unfavorable” outcome. The patient’s features were age, sex, and etiology of the coma. As sex and coma etiology are categorical attributes, we transformed them into numerical variables using the One-Hot-Encoding technique. This transformation resulted in a total of 14 patient features, besides the 100 features extracted from the EEG signals.

Table 5 presents the results obtained for the eight models, including the proposed CNN-LSTM-GA model. The mean and standard deviation of the results obtained in the 10 runs of the GA are also presented. The evaluation is based on the test set, using a dataset division with 80% of the data in the training set (40% training and 40% validation) and 20% in the test set (hold-out). Additionally, the table provides the mean and standard deviation obtained in stratified five-fold cross-validation (CV). These metrics were calculated using the results obtained from the test set of each fold. Results in terms of other complementary metrics, like Geometric Mean, can be seen in Table A4 of Appendix A.

**Figure 5:**
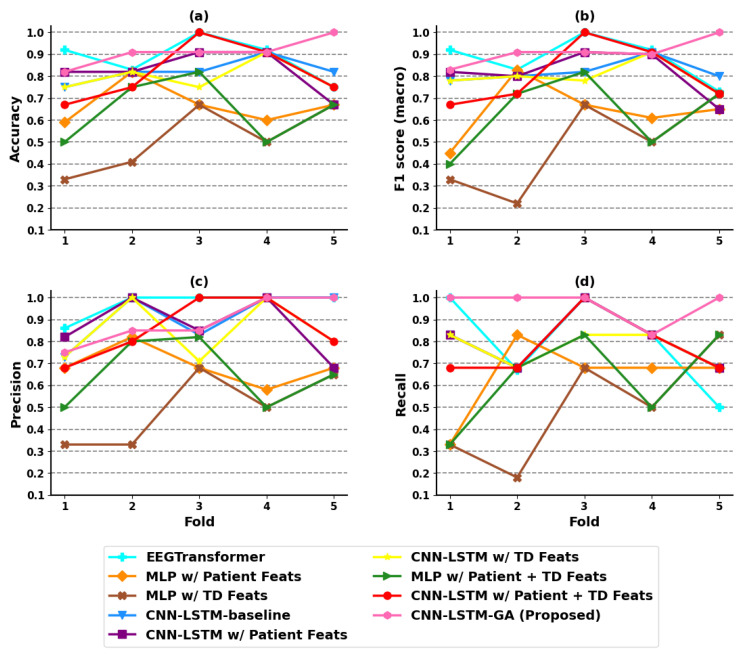
Evaluation for each fold in the coma outcome classification experiment. (**a**) Accuracy; (**b**) F1 score (macro); (**c**) precision; (**d**) recall.

According to Table 5, *MLP w/Patient + TD Feats*, *CNN-LSTM w/Patient Feats* and *CNN-LSTM-GA (Proposed)* obtained the best results for the hold-out experiments. On the other hand, the *CNN-LSTM-GA (Proposed)* obtained the best predictive performance for CV, with an F1(macro) of 0.922±0.046 for the best GA run. Figure 5 shows the results of each model, considering each evaluation metric in each fold of CV, which shows the robust predictive performance of our CNN-LSTM-GA model.

By analyzing the results of the techniques that learn from multimodalities, opposite behavior can be noticed. In the hold-out validation, the predictive performance of *CNN-LSTM w/Patient Feats* was similar to the results obtained by the *MLP w/Patient + TD Feats* model, which incorporates both the patient and TD features as input, with a slight advantage for MLP in terms of F1(macro). However, the results obtained in CV show that the *CNN-LSTM w/Patient Feats* model outperformed the *MLP w/Patient + TD Feats* considerably in terms of all predictive metrics. The same occurred in relation to other techniques like Random Forest and Support Vector Machine, the results of which are presented in Table A5 of Appendix A.

The performance obtained by the *CNN-LSTM w/Patient Feats* model suggests that incorporating patient features as a new modality for the first dense layer enhances the performance of the *CNN-LSTM-baseline* model. However, when all available features, including those of the patient (age, sex, and etiology of the coma) and those extracted from the EEG signals, were used as additional inputs for the first dense layer in the *CNN-LSTM w/Patient + TD Feats* model, the classifier performance deteriorated. The worse results can be mainly attributed to the significantly higher number of features (114 features) used by the *CNN-LSTM w/Patient + TD Feats* model compared to only 14 features utilized in the *CNN-LSTM w/Patient Feats* model. Additionally, the features extracted from the EEG signals are relatively simple and most of them contribute little to the classification. These facts also help to explain the better results of the model optimized by the GA, which are discussed next.

In Table 5, one can observe that our model, *CNN-LSTM-GA*, obtained the same performance as the *CNN-LSTM w/Patient Feats* model in the hold-out experiments. However, in the CV experiments, *CNN-LSTM-GA* obtained the best results, with an F1(macro) of 0.922 ± 0.046. The model optimized by GA utilized only 38 features as additional inputs; five of them are patient features and thirty-three are features extracted from the EEG signals. This number of features is smaller than that used in *CNN-LSTM w/Patient + TD Feats*. These results suggest that the inclusion of specific multimodal features extracted from the EEG signals, along with patient features, contributed to improving the classifier’s performance. This improvement becomes evident when comparing the mean result of CNN-LSTM-GA presented in Table A3 of Appendix A, against the results obtained by other CNN-LSTM variants. Taking the 10 GA runs, *CNN-LSTM-GA* achieved better averaged results of accuracy, F1(macro) and recall than all other models.

As one of the state-of-the-art methods, the EEGTransformer [25] model has demonstrated good performance in EEG classification tasks. Our proposed CNN-LSTM-GA architecture surpasses this model in both hold-out and cross-validation evaluations. This improvement underscores the effectiveness of integrating hybrid deep learning with multimodal inputs and Genetic Algorithm-based optimization techniques.

Analyzing Table 5 reveals a significant difference between the two model performance evaluation methods: hold-out and cross-validation. This difference arises from how each technique operates. In the hold-out approach, the dataset is initially split into three distinct sets: 40% for training, 40% for validation, and 20% for testing. These first two sets are combined into a single training set on which the model is both trained and validated, and afterwards, the model is evaluated on the separate test set. Conversely, in cross-validation, all available data are combined into a single dataset, which is randomly divided into five folds for cross-validation. The model is iteratively trained on four folds and tested on the remaining fold, cycling through all folds to provide a more stable and reliable performance estimate by reducing variability caused by any single data split.

The experimental results indicate that the GA can select an optimized set of features for the first dense layer of the CNN-LSTM model. In addition, the GA can find a model with superior performance and optimized hyperparameters for solving the problem at hand. Additionally, as shown in Table A3 of Appendix A, the CNN-LSTM-GA average results obtained from all GA runs are better than those of almost all other models under comparison listed in Table 5. This suggests that most of the GA runs were able to find a model that outperformed the reference models.

**Figure 6:**
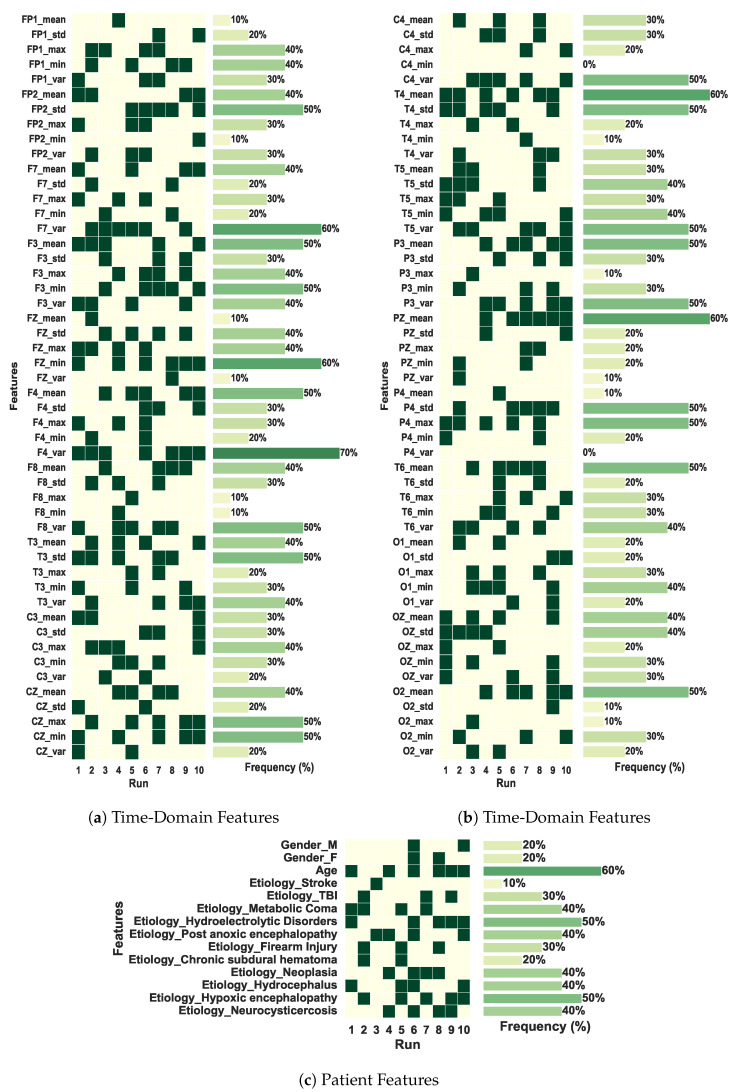
Features selected by the best solution found in each of the 10 runs of the GA in the coma outcome classification experiment. The total of features is 114.

Another interesting analysis is presented in Figure 6, which depicts the most frequent features selected by our multimodal CNN-LSTM-GA architecture, i.e., those ones whose frequency attains at least in 50% of the simulations. Figure 6a,b present results regarding TD features obtained directly from the signals of the electrodes, pointing out that right-side and central electrodes (T4, T6, O2, F4, Fz, Cz) play a relevant role in terms of outcome prediction. Particularly, electrodes T6 and F4 achieve a frequency of 70%, thus pointing out that information tied to the electrical activity related to the cerebral right anterior region is very important for outcome prediction. This result deserves further neurological interpretations and discussions. On the other hand, Figure 6c depicts results regarding another modality related to patient clinical data. One can see that age presents a frequency of 60%, whereas, for hypoxic etiology, frequency attains 50%. This is already expected, since from a medical viewpoint, elderly patients are tied to a minimal recovery ability, and hypoxic phenomena lead to a high rate of patient mortality [6].

### 5.3. Applicability

This investigation has been conducted through a partnership with a Brazilian public hospital. Therefore, we assume that the health units have limited resources, where electroencephalogram equipment is available, but other more sophisticated (and expensive) equipment is not. This assumption is in conformation with most Brazilian health units of the public health system. Specifically, the application of the multimodal architectures proposed in this paper considers the prognosis of patients in coma.

The prognosis of comatose patients admitted to the ICU involves several factors related to coma severity, patient history, demographics, etc. Despite the big challenge behind such a task, the prognosis is crucial to select the most appropriate treatments, as well as to verify their effectiveness. In addition, it can also be helpful in specific cases like brain death, in which clinical protocols aimed at organ donation may be started earlier. In this paper, we found that our CNN-LSTM-GA architecture was able to effectively differentiate patients of favorable and unfavorable outcomes. The results also suggest that other EEG analysis tasks may benefit from our innovative model.

## 6. Conclusions

In this study, we propose a multimodal model defined by the combination of CNN and LSTM networks for the classification of EEG signals related to the prognosis of patients in coma. Our multimodal design allows the combination of EEG signals processed by CNN-LSTM layers with additional modalities, such as those related to patient information. To improve the performance of the classifier, we employ a GA to select the most relevant patient features and extracted EEG features, as well as to optimize the hyperparameters of the hybrid model.

The results obtained from the experiments indicate that incorporating patient features from their clinical records and/or TD features extracted from the EEG signals as additional information in the first dense layer of the CNN-LSTM model enhances the classifier’s performance. However, not all features contribute equally to this improvement. Therefore, utilizing the GA for feature selection enables the creation of a classifier with superior results compared to the several other mono- and multimodal models. Additionally, the GA is effective in finding the optimal CNN-LSTM hybrid architecture for classifying the EEG signals.

Another important contribution is the use of hundreds of EEG data collected from 60 Brazilian patients, which were visually analyzed and selected by expert neurologists to minimize noise in the signals. These procedures are crucial for achieving the statistical power and confidence needed in neurological studies [29]. From a medical standpoint, the results are statistically consistent and promising, warranting further investigation, as noted by the experts who contributed to this article.

The limitations of our study include: (i) the very simple TD features extracted from the EEG signals; and (ii) the dataset size from a deep learning perspective. As our experiments revealed, the extracted features do not contribute significantly to the outcome prediction of patients in coma. To address such a limitation, future works should explore more sophisticated features obtained through signal processing to broaden the scope of analysis. Although our dataset comprises more patients than most related EEG datasets in the literature, expanding its size could further enhance the training of deep learning algorithms. Therefore, we will persist in our efforts to collect additional data for future research.

Regarding future works, the proposed model is general and can be applied to other problems involving the classification of EEG signals, with plans to perform a more detailed statistical analysis to rigorously compare the classification performance across multiple diverse datasets. Therefore, its application to medical problems remains a highly relevant research topic. Additionally, forthcoming studies will explore explainable artificial intelligence (XAI) methods to provide interpretability for the CNN-LSTM model’s decisions. Although some efficient machine learning techniques, such as CNNs, lack intrinsic mechanisms for result explanation, interpretability is essential when applying these models in sensitive domains like medicine and healthcare.

Future efforts will also evaluate advanced optimization algorithms, such as the Coyote Optimization Algorithm, which has demonstrated promising results in various optimization applications. In addition, the effect of demographic variables including sex, age, and coma etiology on the training and validation processes will be evaluated to enhance the robustness of the model. A detailed neurophysiological interpretation and discussion of the electrodes that showed greater relevance in the classification will also be conducted to better understand their significance.

## Figures and Tables

**Table 1:** Comparative analysis of methodologies employed in related works.

Study	DL	HDL	M	FS	HPO
STEADYNet [17]	X				
Xu et al. [12]	X	X			
Ay et al. [10]	X	X			
Lee et al. [11]	X	X			
Pandey et al. [18]	X	X			
Aszemi and Aszemi [19]	X				X
Nikbakht et al. [20]	X				X
Del-Lama et al. [21]	X		X	X	X
Baldo-Júnior et al. [22]	X	X	X		
STRFLNet [24]	X	X	X	X	
EEGTransformer [25]	X				X
Carneiro et al. [27]	X				
CNN-LSTM-GA (ours)	X	X	X	X	X

DL: deep learning; HDL: hybrid deep learning; M: multimodal; FS: feature selection; HPO: hyperparameter optimization.

**Table 2:** Description of our EEG dataset based on the respective number of segments for each coma etiology and outcome.

Etiology	Outcome Prediction	
	Favorable	Unfavorable
Traumatic Brain Injury	170	30
Metabolic Coma	50	130
Stroke	50	70
Post Anoxic Encephalopathy	20	0
Neoplasia	0	20
Hydroelectrolytic Disorders	10	0
Firearm Injury	0	10
Chronic Subdural Hematoma	0	10
Hydrocephalus	0	10
Hypoxic Encephalopathy	0	10
Neurocysticercosis	0	10
Total	300	300

**Table 3:** Confusion matrix example for binary classification.

True Class	Predicted Positive	Predicted Negative
Class A	true positive (TP)	false negative (FN)
Class B	false positive (FP)	true negative (TN)

**Table 4:** Performance metrics and equations.

Metric	Equation
Accuracy	$\frac{TP+TN}{TP+FN+FP+TN}$
Precision	$\frac{TP}{TP+FP}$
Recall (Sensitivity)	$\frac{TP}{TP+FN}$
F1-Score	$\frac{2\times \mathrm{precision}\times \mathrm{recall}}{\mathrm{precision}+\mathrm{recall}}$

**Table 5:** The results obtained by the models for predicting outcome of the coma, including the best model identified by the GA, as well as the average results for all 10 runs. The best results for each metric are in bold.

Model	Validation	Accuracy	F1 (Macro)	Precision	Recall
EEGTransformer [25]	Hold-out	0.75	0.75	0.80	0.67
	CV	0.88 ± 0.09	0.88 ± 0.09	**0.97 ± 0.06**	0.80 ± 0.19
MLP w/Patient Feats	Hold-out	0.75	0.77	0.71	0.83
	CV	0.67 ± 0.09	0.65 ± 0.13	0.68 ± 0.09	0.63 ± 0.16
MLP w/TD Feats	Hold-out	0.58	0.62	0.57	0.68
	CV	0.52 ± 0.13	0.49 ± 0.19	0.49 ± 0.14	0.50 ± 0.24
CNN-LSTM-baseline	Hold-out	0.75	0.73	0.80	0.67
	CV	0.83 ± 0.05	0.82 ± 0.05	0.91 ± 0.12	0.77 ± 0.08
CNN-LSTM w/Patient Feats	Hold-out	**0.83**	0.83	**0.83**	0.83
	CV	0.83 ± 0.09	0.83 ± 0.09	0.87 ± 0.12	0.80 ± 0.13
CNN-LSTM w/TD Feats	Hold-out	0.75	0.73	0.80	0.67
	CV	0.80 ± 0.07	0.80 ± 0.06	0.85 ± 0.13	0.77 ± 0.08
MLP w/Patient + TD Feats	Hold-out	**0.83**	**0.86**	0.75	**1.00**
	CV	0.65 ± 0.13	0.64 ± 0.16	0.65 ± 0.14	0.63 ± 0.19
CNN-LSTM w/Patient + TD Feats	Hold-out	0.75	0.73	0.80	0.67
	CV	0.82 ± 0.12	0.81 ± 0.13	0.85 ± 0.13	0.77 ± 0.13
CNN-LSTM-GA (Proposed)	Hold-out	**0.83**	0.83	**0.83**	0.83
	CV	**0.92 ± 0.05**	**0.92 ± 0.05**	0.89 ± 0.10	**0.97 ± 0.07**

## Data Availability

Data may be made available upon request due to privacy restrictions.

## Appendix A

This is the Appendix of the paper entitled “A Bioinspired Multimodal CNN-LSTM Network for EEG Analysis of Patients in Coma”, submitted to *Sensors*. The document is organized as follows: Appendix A.1 presents an overview about the hyperparameters considered by our CNN-LSTM-GA encoding; Appendix A.2 shows the optimal configuration of hyperparameters found by model; Appendix A.3 presents the results obtained by CNN-LSTM-GA in each one of the 10 GA runs; Appendix A.4 presents experimental results in terms of complementary metrics; and Appendix A.5 shows results obtained by other mono- and multimodal techniques.

### Appendix A.1. Hyperparameters Encoding in CNN-LSTM-GA

Table A1 shows the parameters that can be selected by the GA at each position of the designed encoding.

**Table A1:** Range of values that can be adopted in each encoding position of a GA individual.

Description	Parameter	Values	Type
Activation function of convolutional layers	activation_CNN_layers	[‘relu’,‘tanh’]	string
Activation function of dense layers	activation_Dense_layers	[‘relu’,‘tanh’]	string
Dropout rate	dropout	${\left[0.0,0.2,0.3,0.4,0.5\right]}^{★}$	float
Number of neurons in lstm layer	lstm_units	${\left[0,8,16,32,64\right]}^{★}$	integer
Number of neurons in dense layer	dense_units	${\left[0,32,64,128,256\right]}^{★}$	integer
The number of output filters in the convolution	filter	${\left[0,32,64,128,256\right]}^{★}$	integer
The length of the 1D convolution window	kernel_size	[2,6,9,13]	integer
Number of epochs to train the model	epochs	[5,10,15,20]	integer
Optimizer used for training the model	optimizer	$\left[‘{\mathrm{adam}}^{\prime },‘\mathrm{sgd}’\right]$	string
Learning rate	learning_rate	[0.0001,0.001,0.01,0.05,0.1]	float
Scaling method for the features	scaler	[*‘standard’*, *‘minmax’*]	string
Momentum	momentum	[0.3,0.5,0.7,0.9]	float
Refers to adding additional values at the beginning and/or end of a 1D input sequence to preserve its length and/or prevent information loss	padding	[‘same’, ‘causal’]	string
Size of the max pooling window	pool_size	${\left[0,2,3,4,5\right]}^{★}$	integer
The corresponding feature is selected (True) or not (False). There is an element of the chromosome for each feature.	feature	[*’true’*, *’false’*]	boolean

^★^ A value of 0 indicates the absence of the respective layer.

### Appendix A.2. Optimal Configuration of Hyperparameters for CNN-LSTM-GA

Table A2 presents the optimized architecture and hyperparameters of the CNN-LSTM-GA model in the coma outcome classification experiment. The model was trained using the *binary_accuracy* metric function and the loss *binary_crossentropy* function.

**Table A2:** Architecture and parameters of the proposed CNN-LSTM-GA model in the coma outcome classification experiment. The model was trained for 20 epochs, using the Adam optimizer with *lr* = 0.001 and the external features data was normalized using standard scaling. The learning and architecture parameters were found by the GA.

No.	Layer	Hyperparameters
0	Input	[signal, 20]
1	Conv1D	filters = 32, kernel_size = 6, padding = *same*, activation = *TanH*
2	Dropout	rate = 0.5
3	Conv1D	filters = 256, kernel_size = 2, padding = *same*, activation = *TanH*
4	MaxPooling1D	pool_size = 5, strides = 5
5	Dropout	rate = 0.2
6	MaxPooling1D	pool_size = 5, strides = 5
7	Dropout	rate = 0.5
8	Conv1D	filters = 128, kernel_size = 2, padding = *causal*, activation = *TanH*
9	MaxPooling1D	pool_size = 2, strides = 2
10	Dropout	rate = 0.5
11	Dropout	rate = 0.5
12	LSTM	units = 16, return_sequences = True
13	Dropout	rate = 0.3
14	LSTM	units = 16, return_sequences = True
15	GlobalAveragePooling1D	-
16	Flatten	-
17	Concatenate	(Flatten output + 38 External features)
18	Dense	units = 32, activation = *TanH*
19	Dropout	rate = 0.2
20	Dense	units = 1, activation = *Sigmoid*

### Appendix A.3. CNN-LSTM-GA Results

Table A3 shows the results obtained by the CNN-LSTM-GA in each of the 10 runs of the GA for the coma outcome classification task.

**Table A3:** Results obtained by the CNN-LSTM-GA in each of the 10 runs of the GA in the coma outcome classification experiment. The results from the best model are in bold.

Run	Dataset	Accuracy	F1 Score (Macro)	Precision	Recall
1	80% Train/20% Test	0.833	0.833	0.833	0.833
	Cross-Validation	0.917 ± 0.075	0.913 ± 0.079	0.950 ± 0.100	0.900 ± 0.133
2	80% Train/20% Test	0.750	0.727	0.800	0.677
	Cross-Validation	0.867 ± 0.041	0.855 ± 0.049	0.950 ± 0.100	0.800 ± 0.125
3	80% Train/20% Test	0.750	0.727	0.800	0.667
	Cross-Validation	0.867 ± 0.041	0.855 ± 0.049	0.950 ± 0.100	0.800 ± 0.125
4	80% Train/20% Test	0.750	0.727	0.800	0.667
	Cross-Validation	0.833 ± 0.085	0.886 ± 0.077	0.905 ± 0.131	0.900 ± 0.133
5	80% Train/20% Test	0.750	0.727	0.800	0.667
	Cross-Validation	0.833 ± 0.041	0.868 ± 0.056	0.971 ± 0.057	0.800 ± 0.125
6	80% Train/20% Test	0.833	0.833	0.833	0.833
	Cross-Validation	0.867 ± 0.067	0.865 ± 0.073	0.881 ± 0.103	0.867 ± 0.125
7	80% Train/20% Test	0.750	0.727	0.800	0.667
	Cross-Validation	0.833 ± 0.041	0.868 ± 0.056	0.971 ± 0.057	0.800 ± 0.125
8	80% Train/20% Test	0.833	0.833	0.833	0.833
	Cross-Validation	0.833 ± 0.067	0.876 ± 0.077	0.921 ± 0.102	0.867 ± 0.163
9	80% Train/20% Test	**0.833**	**0.833**	**0.833**	**0.833**
	Cross-Validation	**0.917 ± 0.053**	**0.922 ± 0.046**	**0.893 ± 0.096**	**0.967 ± 0.067**
10	80% Train/20% Test	0.750	0.727	0.800	0.667
	Cross-Validation	0.850 ± 0.082	0.857 ± 0.079	0.843 ± 0.129	0.900 ± 0.133
Mean	80% Train/20% Test	0.783 ± 0.043	0.769 ± 0.055	0.813 ± 0.017	0.733 ± 0.086
	Cross-Validation	0.877 ± 0.026	0.877 ± 0.024	0.924 ± 0.042	0.860 ± 0.058

### Appendix A.4. Stratified 5-Fold Cross-Validation Results with Complementary Metrics

Table A4 presents the results obtained in the coma outcome classification experiment for mono- and multimodal models in stratified five-fold cross-validation, including the CNN-LSTM-GA. The table provides the following metrics based on stratified five-fold cross-validation: mean, standard deviation, Geometric Mean (G-mean), maximum, and minimum values. These metrics were calculated using the results obtained from the test set of each fold.

**Table A4:** Results obtained by the mono- and multimodal models for coma outcome classification considering a stratified 5-fold cross-validation.

Model	Cross Validation	Accuracy	F1 Score (Macro)	Precision	Recall
EEGTransformer [25]	Mean and std	0.88±0.09	0.88±0.09	**0.97±0.06**	0.80±0.19
	Max	1.00	1.00	1.00	1.00
	G-Mean	0.88	0.87	0.97	0.77
	Min	0.75	0.73	0.86	0.50
MLP w/Patient Feats	Mean and std	0.67 ± 0.09	0.65 ± 0.13	0.68 ± 0.09	0.63 ± 0.16
	Max	0.83	0.83	0.83	0.83
	G-Mean	0.66	0.63	0.68	0.61
	Min	0.58	0.44	0.57	0.33
MLP w/TD Feats	Mean and std	0.52 ± 0.13	0.49 ± 0.19	0.49 ± 0.14	0.50 ± 0.24
	Max	0.67	0.71	0.67	0.83
	G-Mean	0.50	0.45	0.47	0.43
	Min	0.33	0.22	0.33	0.17
CNN-LSTM-baseline	Mean and std	0.83 ± 0.05	0.82 ± 0.05	0.91 ± 0.12	0.77 ± 0.08
	Max	0.92	0.91	1.00	0.83
	G-Mean	0.83	0.82	0.90	0.76
	Min	0.75	0.77	0.71	0.67
CNN-LSTM w/Patient Feats	Mean and std	0.83 ± 0.09	0.83 ± 0.09	0.87 ± 0.12	0.80 ± 0.13
	Max	0.92	0.92	1.00	1.00
	G-Mean	0.83	0.82	0.86	0.79
	Min	0.67	0.68	0.68	0.68
CNN-LSTM w/TD Feats	Mean and std	0.80 ± 0.07	0.80 ± 0.06	0.85 ± 0.13	0.77 ± 0.08
	Max	0.92	0.91	1.00	0.83
	G-Mean	0.80	0.79	0.84	0.76
	Min	0.75	0.73	0.71	0.67
MLP w/Patient + TD Feats	Mean and std	0.65 ± 0.13	0.64 ± 0.16	0.65 ± 0.14	0.63 ± 0.19
	Max	0.83	0.83	0.83	0.83
	G-Mean	0.64	0.61	0.64	0.60
	Min	0.50	0.40	0.50	0.33
CNN-LSTM w/Patient + TD Feats	Mean and std	0.82 ± 0.12	0.81 ± 0.13	0.85 ± 0.13	0.77 ± 0.13
	Max	1.00	1.00	1.00	1.00
	G-Mean	0.81	0.80	0.84	0.76
	Min	0.67	0.67	0.67	0.67
CNN-LSTM-GA (Proposed)	Mean and std	0.92 ± 0.05	0.92 ± 0.05	0.89 ± 0.10	0.97 ± 0.07
	Max	1.00	1.00	1.00	1.00
	G-Mean	0.92	0.92	0.89	0.96
	Min	0.83	0.86	0.75	0.83

### Appendix A.5. Results of Other Mono- and Multimodal Models

Table A5 presents the results obtained in the coma outcome classification experiment for the Random Forest and Support Vector Machine (SVM) in stratified five-fold cross-validation. The table provides the following metrics based on stratified five-fold cross-validation: mean, standard deviation, Geometric Mean (G-mean), maximum, and minimum values. These metrics were calculated using the results obtained from the test set of each fold.

**Table A5:** The results obtained by other mono and multimodal models for predicting coma outcome in stratified 5-fold cross-validation.

Model	Cross Validation	Accuracy	F1 Score (Macro)	Precision	Recall
Random Forest w/Patient Feats	Mean and std	0.72 ± 0.09	0.68 ± 0.14	0.74 ± 0.06	0.67 ± 0.21
	Max	0.83	0.86	0.80	1.00
	G-Mean	0.71	0.67	0.73	0.63
	Min	0.58	0.44	0.67	0.33
Random Forest w/TD Feats	Mean and std	0.48 ± 0.08	0.50 ± 0.12	0.48 ± 0.08	0.53 ± 0.19
	Max	0.58	0.63	0.57	0.83
	G-Mean	0.48	0.48	0.47	0.50
	Min	0.33	0.33	0.33	0.33
Random Forest w/Patient + TD Feats	Mean and std	0.60 ± 0.10	0.55 ± 0.13	0.62 ± 0.13	0.50 ± 0.15
	Max	0.75	0.73	0.80	0.67
	G-Mean	0.59	0.53	0.61	0.48
	Min	0.50	0.40	0.50	0.33
SVM w/Patient Feats	Mean and std	0.65 ± 0.10	0.65 ± 0.09	0.67 ± 0.12	0.63 ± 0.07
	Max	0.75	0.73	0.80	0.67
	G-Mean	0.64	0.64	0.66	0.63
	Min	0.50	0.50	0.50	0.50
SVM w/TD Feats	Mean and std	0.42 ± 0.12	0.45 ± 0.18	0.40 ± 0.12	0.53 ± 0.29
	Max	0.58	0.71	0.55	1.00
	G-Mean	0.40	0.41	0.38	0.45
	Min	0.25	0.18	0.20	0.17
SVM w/Patient + TD Feats	Mean and std	0.62 ± 0.10	0.61 ± 0.08	0.64 ± 0.13	0.60 ± 0.08
	Max	0.75	0.73	0.80	0.67
	G-Mean	0.61	0.61	0.63	0.59
	Min	0.50	0.50	0.50	0.50
